# Synergy of Linezolid with Several Antimicrobial Agents against Linezolid-Methicillin-Resistant Staphylococcal Strains

**DOI:** 10.3390/antibiotics9080496

**Published:** 2020-08-09

**Authors:** María-José Valderrama, María Alfaro, Icíar Rodríguez-Avial, Elvira Baos, Carmen Rodríguez-Avial, Esther Culebras

**Affiliations:** 1 Departamento de Genética, Fisiología y Microbiología, Universidad Complutense de Madrid, 28040 Madrid, Spain; 2 Hospital Clínico San Carlos, 28040 Madrid, Spain; mariaalfarosierra@gmail.com (M.A.); iciar.rodriguezavial@salud.madrid.org (I.R.-A.); elvira.baos@salud.madrid.org (E.B.); esther.culebras@salud.madrid.org (E.C.); 3 Departamento de Medicina, Universidad Complutense de Madrid, 28040 Madrid, Spain; cravial@med.ucm.es

**Keywords:** *Staphylococcus* spp., linezolid resistance, plazomicin, synergy

## Abstract

Linezolid is a synthetic oxazolydinone active against multi-resistant Gram-positive cocci that inhibits proteins synthesis by interacting with the 50S ribosomal subunit. Although linezolid-resistant strains are infrequent, several outbreaks have been recently described, associated with prolonged treatment with the antibiotic. As an alternative to monotherapy, the combination of different antibiotics is a commonly used option to prevent the selection of resistant strains. In this work, we evaluated combinations of linezolid with classic and new aminoglycosides (amikacin, gentamicin and plazomicin), carbapenems (doripenem, imipenem and meropenem) and fosfomycin on several linezolid- and methicillin-resistant strains of *Staphylococcus aureus* and *S. epidermidis,* isolated in a hospital intensive care unit in Madrid, Spain. Using checkerboard and time-kill assays, interesting synergistic effects were encountered for the combination of linezolid with imipenem in all the staphylococcal strains, and for linezolid–doripenem in *S.epidermidis* isolates. The combination of plazomicin seemed to also have a good synergistic or partially synergistic activity against most of the isolates. None of the combinations assayed showed an antagonistic effect.

## 1. Introduction

Antibiotic resistance is considered a major global public health problem and constitutes a challenge for the treatment of infections caused by multi-drug-resistant microorganisms. Gram-positive bacteria are responsible for a high proportion of community- and hospital-acquired invasive infections, and *Staphylococcus aureus*, enterococci and coagulase-negative staphylococci are the most frequently isolated agents [1]. Although the rates of bacteremia caused by methicillin-resistant *S. aureus* (MRSA) have remained constant or have decreased over the last few years, the emergence of new isolates of *S. epidermidis* resistant, or with reduced sensitivities, to beta lactams or non-beta-lactam antibiotics such as glycopeptides (e.g., vancomycin) and aminoglycosides (e.g., gentamicin) causes severe difficulties or failures in patient treatments [2,3].

Linezolid is a synthetic oxazolidinone active against multi-resistant Gram-positive cocci and is one of the recommended treatments for pneumonia, bacteremia or central nervous system and soft tissue infections [4]. Linezolid inhibits protein synthesis during the initiation of translocation by interacting with the peptidyl transferase center of the 50S ribosomal subunit [5]. It shows good pharmacokinetic/pharmacodynamical properties and excellent oral bioavailability, and it appears to reduce biofilm formation by staphylococci [6,7]. Although it was demonstrated that the resistance to linezolid occurred rarely at a frequency of <1 resistant mutant per 8 × 10^11^ colony forming units (CFU) [8], soon after its approval for clinical use, several outbreaks of linezolid-resistant isolates were described [9]. Linezolid resistance is based on mutations in several 23S rRNA positions, including conserved nucleotides at the antibiotic binding pocket and other nucleotides, and the mutation Gly2576Thr is the most frequent one. Additionally, deletions or mutations in ribosomal proteins L3 and L4 can cause decreased susceptibility to linezolid. Finally, the only transferable mechanism of linezolid resistance is the plasmidic *cfr* gene. It encodes a methyltransferase that methylates a specific nucleotide in the binding site at the 23S rRNA [5].

Failures in the treatment of multi-resistant staphylococci infections with classical or new antibiotics in monotherapy has led to the recommendation of combined therapies, mainly based on empirical clinical experience [4,10]. Combinations of antibiotics are sought to avoid the emergence of resistances, enhance activity or reduce the doses and/or duration of drug administration [11]. Some experimental studies have been published on combinations of linezolid with different antibiotics of several families (glycopeptides, aminoglycosides, rifamycins or quinolones) against *Staphylococcus aureus*. Nevertheless, many strains were susceptible to linezolid and susceptible to the rest of antibiotics assayed in combination [12,13], and, additionally, there exist very few data for coagulase-negative staphylococci such as *S. epidermidis.*

In this work, we studied several linezolid-resistant *S. aureus* strains, isolated during the first European nosocomial outbreak in an intensive care unit (ICU) in Madrid, Spain [14], and *S. epidermidis* strains, also isolated in the ICU four years later. The main objective was to evaluate different combinations of linezolid with several antibiotics with different activities—such as classic and new aminoglycosides, which inhibit protein synthesis, and carbapenems and fosfomycin, which interact with cell wall formation—with the aim of providing experimental data that could help with the selection of new options for treating infections caused by linezolid-resistant staphylococci.

## 2. Results

### 2.1. Antimicrobial Susceptibility Testing

The susceptibility of the strains was studied by broth microdilution, and the minimum inhibitory concentration (MIC) values are shown in Table 1. *S. aureus* strains were resistant to gentamicin and fosfomycin and showed elevated MIC values for carbapenems (ranging from 64 to 128 mg/mL for doripenem, imipenem and meropenem).

Fosfomycin showed good in vitro activity against *S. epidermidis* (MIC values 1–2 mg/L), while the isolates had elevated MICs for meropenem (8–16 mg/L), although they were lower than those of *S. aureus* (128 mg/L). The FDA breakpoint for plazomicin was used (2 mg/L), and all the staphylococcal isolates presented lower MIC values (0.0625–0.25 mg/L), as would be reasonable, since plazomicin has not been approved yet in European countries. Nevertheless, the studied *S. epidermidis* strains were resistant to amikacin and gentamicin (except HCSC-Se12, which was sensible to amikacin, with a MIC of 8 mg/mL, just at the breakpoint).

### 2.2. Checkerboard Results

The results of the study of combinations of linezolid with different antibiotics are shown in Table 2, which include the minimum fractional inhibitory concentration index for each combination as well as the concentrations of both antibiotics in the synergistic points. The best combination corresponded to linezolid plus imipenem, as synergistic effects were encountered for all isolates of *S. aureus* and *S. epidermidis* (partial synergy, PS, in *S. aureus* HCSC-Sa3). All the strains of *S. epidermidis* were adequately inhibited in vitro with the combination linezolid–doripenem, while this combination was less effective against *S. aureus* as various results were obtained (one synergy (S), three PS or one indifferent (I)). The third combination of linezolid with carbapenems (linezolid–meropenem) rendered less satisfactory results as PS was observed for most of the strains, except for three *S. epidermidis* isolates, where synergy was observed. The results of the combination fosfomycin plus linezolid were not homogeneous among the staphylococcal isolates. Among the aminoglycosides tested in combination, plazomicin seemed to have synergistic (four strains), or partially synergistic (five strains), effects in most of the staphylococcal isolates, while for gentamicin and amikacin, mostly PS or I, respectively, was observed. Finally, no antagonism was found for any of the strains and antibiotic combinations.

The MIC values of each antibiotic in combination at which a synergistic effect was obtained are also shown in Table 2. As observed, some of the combinations are synergistic with adequate concentrations of the antibiotics (marked with asterisks in Table 2), as the concentrations used are lower than the SSCs. Considering only the potentially useful combinations, linezolid plus imipenem would still be the best option for *S. epidermidis*, followed by the combinations with doripenem, meropenem and fosfomycin. Nevertheless, for the *S. aureus* strains, useful combinations of linezolid are scarce.

### 2.3. Time-Kill Curves

Time-kill curves were for generated for eight strains (four *S. aureus* and four *S. epidermidis*) using 1/2 of the MIC of linezolid for each strain and a fixed concentration, SSC, for the other antibiotic in each combination. The results, expressed as CFU/mL after 24 h of incubation, are shown in Table 3. Several synergistic combinations of linezolid were obtained, particularly for the *S. epidermidis* strains. In general, the best combinations were linezolid–carbapenems (meropenem and imipenem) for all the staphylococcal isolates assayed, as also shown in Table 2. Some of them are coincident with the results obtained in the checkerboard assay (Table 2 and Table 3, marked with asterisks), and more synergistic combinations were found as new concentrations were used.

**Figure 1:**
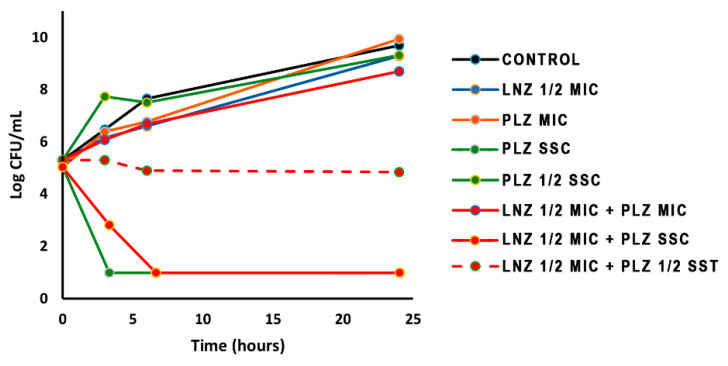
Time kill curves of LNZ (1/2 MIC) plus different concentrations of PLZ against *S. aureus* HCSC-Sa14. LNZ: linezolid; PLZ: plazomicin; MIC: minimum inhibitory concentration; SSC: steady state concentration; CONTROL: growth in Mueller–Hinton broth without antibiotics.

No synergy was observed for any of the isolates when linezolid was combined with SSC plazomicin (10 mg/L), as this concentration is higher than the MIC (0.125–0.25 mg/L, Table 2). Different concentrations of plazomicin combined with 1/2 MIC linezolid were then assayed for a representative strain, and the results are represented in Figure 1, as CFU/mL vs. time, in comparison with the growth of the strain without antibiotics. Growth was slightly retarded at 6 h of incubation, but not inhibited at 24 h, in the presence of a subinhibitory concentration of linezolid (1/2 MIC), and it was not inhibited with a MIC (0.25 mg/L, Table 1) or even 1/2 SSC (5 mg/L) of plazomicin. Plazomicin at 10 mg/L (SSC) alone or in combination with 16 mg/L of linezolid (1/2 MIC) rapidly killed the bacteria, as expected. Nevertheless, a clear inhibitory process was observed when combining 1/2 MIC linezolid and 1/2 SSC plazomicin, thus indicating a good combination of the antibiotics at these concentrations.

## 3. Discussion

Resistance to linezolid is still infrequent in terms of epidemiological incidence, remaining below 0.1% among Gram-positive cocci [5,15] and reaching 0.8% in coagulase-negative staphylococci [16]. Nevertheless, important outbreaks due to linezolid-resistant strains have been reported worldwide, with the highest rates in the United States, Brazil, Europe, India and Japan [5,9,14]. The most frequent mechanism of resistance to linezolid is the mutation Gly2576Thr in the V domain of 23S rRNA (in approximately 60% of *S. aureus* and coagulase-negative staphylococci), followed by the presence of the *cfr* gene (54% of *S. aureus*/15% of coagulase-negative species) and L3 and L4 mutations (20% of *S. aureus*/35% of coagulase-negative staphylococci) [9].

The five *S. aureus* strains included in this study (100%) showed two resistance mechanisms, the *cfr* gene and L3 mutation (Table 4). The strains were isolated from intensive care unit patients and belonged to a single clone [14]. Of the five *S. epidermidis* isolates, only one (20%) harbored the *cfr* gene, in accordance with the low rates of *cfr* coagulase-negative isolates encountered in a meta-analysis of linezolid-resistant staphylococci [9]. *cfr* is a plasmidic gene that is transferable horizontally, and it could be hypothesized that normal microbiota, i.e., *S. epidermidis*, could have the potential to transfer the *cfr* gene to more pathogenic staphylococci such as *S. aureus* [17]. In the present study, the mutation Gly2576Thr in 23S rRNA was detected in two *S. epidermidis* isolates (40%) but not in *S. aureus*. Interestingly, one of these strains, HCSC-Se12, presented the mutation in five copies of its rRNA, besides the *cfr* gene, and this could be responsible for its very high MIC of linezolid (256 mg/L), as has been previously described [1,18]. A mutation in the L4 ribosomal protein is rarely described in *S. aureus,* although it is detected in approximately 20% of coagulase-negative strains [9]. Similarly, an Asn158Ser L4 mutation was encountered in one of our *S. epidermidis* isolates (20%). This strain, HCSC-Se45, had two resistance determinants (L4 and L3 mutations), and this could be related to the increased MIC of linezolid (64 mg/L, compared with the average MIC of 16 mg/L for the strains with a single mechanism), as reported [5]. According to the literature, exposure to linezolid in treated patients is the cause of the selection of resistant mutants among staphylococci (rRNA, L3 and L4 modifications) [5,19] or enterococci [20], due to antibiotic pressure. This could be the situation of the resistant strains of *S. aureus* and *S. epidermidis* included in this study, which were all isolated from ICU patients treated with linezolid [14].

Besides linezolid, seven antibiotics of different families were included in the study. Not only the strains of *S. aureus* but also *S. epidermidis* isolates were highly resistant, thus representing an important problem for the treatment of the patients [14,22]. The in vitro activity of plazomicin, a novel aminoglycoside recently approved in the USA but not yet in European countries, was analyzed. Although, to date, some resistant enterococci or *Streptococcus pneumoniae* have been described, plazomicin shows good activity against *S. aureus*, including MRSA, and coagulase-negative staphylococci [23]. Similarly, all the strains included in this study were susceptible to plazomicin, with low MIC values (Table 1).

The possibilities for the successful treatment of infections caused by multi-resistant staphylococci are certainly scarce, although new antibiotics such as linezolid, daptomycin, ceftobriol and dalvamycin are good or promising alternatives [16]. The emergence of nosocomial linezolid-resistant strains of *S. aureus* and coagulase-negative staphylococci, such as *S. epidermidis*, is worrying due to the high capacity for the adaptation of staphylococci, the possibility of the transmission of the *cfr* gene and the selection of mutants under antibiotic selective pressure [19,24].

In clinical practice, the use of combined therapies is based on empirical experience, and it is usually established to cover multi-resistant strains or polymicrobial infections before a laboratory confirmation of the specific agents and the susceptibility profiles. The in vitro studies of combinations of antibiotics are certainly necessary to provide data, based on synergistic activities, to support combined therapies. Unfortunately, several studies have described unsuccessful combinations of linezolid with different families of antibiotics for *S. aureus* resistant or susceptible to methicillin: indifference was mostly found for the combinations with rifampicin [25,26,27], quinolones (ciprofloxacin, levofloxacin and morifloxacin) [26,27] or some protein synthesis inhibitors (clindamycin, eritromycin, tetramycin, and gentamicin) [13,25,28,29]. Vancomycin and linezolid were also indifferent or antagonistic for a high proportion of the MRSA or MSSA (methicillin-susceptible *S. aureus*) strains tested [26,30]. In this work, we analyzed the combination of linezolid with aminoglycosides in 10 strains of methicillin-resistant staphylococci (five *S. aureus* and five *S. epidermidis*), and we found similar unsatisfactory results for amikacin and gentamicin (Table 2). Plazomicin is a new-generation aminoglycoside with enhanced activity, alone or in-combination, against multi-drug-resistant microorganisms, including MRSA [31]. We found good results when combining linezolid with plazomicin, as synergy or partial synergy occurred in 9/10 of the stains (40% S; 50% PS). We only found one study of the combination of linezolid and plazomicin, in which 13% of synergistic results were reported using plazomicin- and linezolid-susceptible staphylococcal strains [32]. As mentioned, the isolates used in our study were resistant to linezolid (Table 1), so the combination with plazomicin, although it is not still in use in Europe, could be a good option for the treatment of multi-drug-resistant staphylococci.

We also found a remarkable synergistic effect when linezolid was combined with antibiotics that inhibit cell wall synthesis, fosfomycin and carbapenems, in 50% (for fosfomycin) to 90% (for imipenem) of the strains assayed (Table 2). Several studies have shown very diverse synergistic associations in 10–90% of *S. aureus* isolates [12,25,29,33,34,35,36,37]. In these reports, all the strains were susceptible to linezolid and most of them were susceptible to the second antibiotic employed (fosfomycin or imipenem), and consequently, they could be used in monotherapy at normal clinical doses or in combination at reduced concentrations. Nevertheless, the MRSA of our study were resistant to linezolid and fosfomycin and showed high MIC values for the carbapenems (Table 1). However, more interestingly, the three combinations (with imipenem, doripenem and fosfomycin) did inhibit bacterial growth at concentrations inferior to the CMIs, and the best association was linezolid with imipenem (the individual CMIs were 32 and 128 mg/L, respectively) (Table 2 and Table 3). Therefore, it could be inferred that these antibiotics could be used in combination for treatment when monotherapy is not possible.

It is worth highlighting the results obtained in this work for *Staphylococcus epidermidis* because, as discussed before, most of the studies on the combinations of linezolid are focused on *S. aureus*, with few data about *S. epidermidis*. In two studies, linezolid-susceptible and resistant strains were used (12 susceptible, 6 resistant) and indifferent effects were described for the combinations with ceftobiprole, rifampicin and clindamycin [38,39]. Synergy was only found with fosfomycin in two strains [33]. We found good synergistic combinations for all or several *S. epidermidis* isolates when linezolid was associated with carbapenems, fosfomycin and aminoglycosides (Table 2).

Based on the pharmacokinetic characteristics of each antibiotic, adequate concentrations should be reached and maintained in the plasma and tissues according to the therapeutic regime (SSC, steady state concentration), in order to inhibit bacterial growth without causing toxicity [40]. Therefore, several potential synergistic combinations could not be used in vivo because the concentrations of one or two antibiotics were higher than the SSCs. In Table 2, the potentially useful combinations are marked with asterisks, and they are almost 50% of the total synergistic data. In order to better confirm these results, time-kill curves were generated at fixed concentrations that could be reached in plasma (Table 3). In both studies, the best results were obtained for the combinations of linezolid with carbapenems. Notably, the growth of the strain HCSC-Se45 was very efficiently inhibited (more than 2 log CFU/mL, Table 2) by the combination of linezolid with all the antibiotics (except plazomicin). Nevertheless, it should be noted that the concentration of linezolid used (125 mg/mL) was higher than its SSC (16 mg/mL), and consequently, this combination would not have clinical utility. It could be concluded that the combinations of linezolid with imipenem or meropenem could be useful for the clinical treatment of staphylococcal infections, as they were synergistic in vitro for linezolid-susceptible strains [29,34] and resistant isolates (this study).

Finally, the importance of the methods used to study the antibiotic combinations should be highlighted [11]. Checkerboard is a simple methodology that allows the assay at the same time of different combinations and several concentrations of each compound, and the results offer a good approximation of the possibilities of synergism among antibiotics. Time-kill curves are laborious and time-consuming to generate, but they reveal bacterial growth inhibition (and regrowth in some cases) or bactericidal effects. They are also very useful for confirming the initial data of fractional inhibitory concentrations (FICs) or detecting more synergistic combinations at different concentrations that could be reached in the plasma (Table 3 and Figure 1). Sometimes, the rates of synergism found with checkerboard techniques are lower than those determined with time-kill curves [11], as shown in Table 2 and Table 3 for linezolid + meropenem, as an example. Moreover, some authors claim a severe lack of correlation between methods [41]. The antibiotic concentrations assayed in time-kill experiments are not homogeneous among different studies, ranging from very low values to multiples of MICs, and sometimes, these concentrations are not reached in body fluids nor at the site of infection [12,25,36]. In our experience, the results obtained with checkerboard are useful for selecting a set of combinations/concentrations of antibiotics to be tested afterwards by time-kill curves, testing more concentrations of clinical significance (SSC) and analyzing the bacteriostatic or bactericidal effects of the antibiotics in combination. In any case, both laboratory methods are static and do not mimic the real in vivo situation, in which the antibiotic concentrations are not constant. To overcome this problem, dynamic in vitro experiments have been designed and in vivo studies using animal models should be considered necessary [11,35,36,42].

Many antimicrobial resistance mechanisms are well characterized and harbored by individual bacterial strains and clonal groups. Based on this and together with the results of susceptibility tests from the laboratory and pharmacological experience, patient treatment can be adequately designed. Nevertheless, the synergistic activity of combined molecules is not always understood [11] and it seems to be strain-dependent, as shown in this work and in the literature. Consequently, it would be desirable to study, in the laboratory, the possibilities of antibiotic associations when combined treatments against multi-drug-resistant strains are necessary. In this work, we have described the synergistic effect of linezolid in combination with several antibiotics such as imipenem, doripenem or meropenem, fosfomycin, and plazomicin against linezolid-resistant *S. aureus* and *S. epidermidis* strains. The results obtained for *S. epidermidis* are of interest, as studies on coagulase-negative staphylococci are still scarce.

## 4. Materials and Methods

### 4.1. Strains

Ten strains of methicillin- and linezolid-resistant staphylococci were included: 5 *S. aureus* and 5 *S. epidermidis*. They were isolated in the intensive care unit (ICU) of the Hospital Clínico San Carlos (HCSC), Madrid, Spain, from different clinical samples (Table 4). Recommended reference strains were included as a control for MIC (minimum inhibitory concentration) and lethality studies [43].

### 4.2. Antimicrobial Agents

The following antimicrobial agents were used: linezolid (Pfizer, Inc., New York, NY, USA), amikacin and gentamicin (purchased from Sigma-Aldrich, Spain), plazomicin (Achaogen, South San Francisco, CA, USA), fosfomycin (ERN Laboratories, Barcelona, Spain) and imipenem, meropenem and doripenem (provided by the hospital pharmacy at HCSC).

### 4.3. Susceptibility Testing

The minimum inhibitory concentration (MIC) of each antibiotic was determined by the microdilution method in Mueller–Hinton broth (Becton Dickinson, Clare, Ireland) [44]. Serial antibiotic dilutions and a control solution were prepared in 96-well microtitre plates (Nalge Nunc International, Roskilde, Denmark). The plates were inoculated with approximately 10^5^ CFU/mL of each bacterial strain to obtain a final volume of 100 µL per well. The MIC was defined as the lowest concentration that prevented growth after 18–20 h of incubation in ambient air at 37 °C. All tests were carried out in duplicate. The results were interpreted using clinical breakpoints as defined by EUCAST (European Committee on Antimicrobial Susceptibility Testing). To date, no breakpoints for plazomicin have been established by EUCAST, so FDA (Food and Drug Administration) values were used [45].

### 4.4. Checkerboard Technique

The checkerboard microdilution method was used to determine the in vitro activity of linezolid combined with amikacin, gentamicin, plazomicin, fosfomycin, imipenem, meropenem and doripenem. The range of drug concentrations used in the assay was such that the dilution range encompassed the MIC for each drug employed in the analysis, the highest concentration being 2× MIC. Serial two-fold dilutions of each antibiotic tested were prepared and mixed in each well of a microtitre plate so that each row (and column) contained a fixed amount of one agent and decreasing amounts of the second agent. The final inoculum was approximately 10^5^ CFU/mL in a 100 μL final volume, and the plates were incubated for 18–20 h at 37 °C. All tests were carried out in duplicate. Fractional inhibitory concentrations (FIC) were calculated for each combination, and the smallest FIC value was used to establish the antimicrobial combination interaction for each specific strain. The interpretation of the FIC index (FICI) was as follows: ≤0.5, synergy; >0.5–1.0, partial synergy; >1.0–4.0, indifference; and antagonism if >4.0. All the procedures were performed according to CLSI (Clinical and Laboratory Standards Institute) [46].

### 4.5. Time-Kill Assays

Tubes containing freshly prepared Mueller–Hinton broth supplemented with the drug were inoculated with the staphylococcal isolates at a density of 10^5^ CFU/mL and incubated in a shaking bath at 37 °C for 24 h. The antibiotic concentration used in the time-kill assays corresponded to 0.5-fold the linezolid MIC values when in combination with the steady state concentrations (SSCs) of the other antimicrobial compounds: amikacin, 32 mg/mL [47]; gentamicin, 10 mg/mL [48]; plazomicin, 10 mg/mL (Achaogen); fosfomycin, 83 mg/mL [49]; imipenem, 8 mg/mL [47]; meropenem, 6,8 mg/mL [49]. Additional curves were performed with one *S. aureus* strain using different concentrations of plazomicin (SST, 1/2 SST, MIC and 1/2 MIC). Samples were taken at 0, 3, 6 and 24 h, serially diluted, spread on Mueller–Hinton agar plates, and incubated at 37 °C for 24 h. The time-kill curves were constructed by plotting mean colony counts (log10 CFU/mL) vs. time. Kill curves and colony counts for each curve were carried out in duplicate. Synergy was interpreted as a ≥2 log10 decrease in CFU/mL by the drug combination when compared with the value from its most active drug alone [11].

## Figures and Tables

**Table 1:** Susceptibility of staphylococcal isolates to linezolid and several antibiotics: plazomicin, amikacin, gentamicin, fosfomycin, meropenem, imipenem and doripenem.

Bacterial Species	Isolate Number	MIC (mg/L) (Breakpoint)							
		LNZ (4)	AMK (8)	GM (1)	PLZ (2)	FOSF (32)	IMP *	MER *	DOR *
*S. aureus*	HCSC-Sa3	32	4	64	0.25	128	128	128	64
*S. aureus*	HCSC-Sa8	32	4	64	0.25	256	128	128	64
*S. aureus*	HCSC-Sa11	32	4	64	0.25	128	128	128	64
*S. aureus*	HCSC-Sa13	32	4	64	0.25	512	128	128	64
*S. aureus*	HCSC-Sa14	32	4	64	0.25	128	128	128	64
*S. epidermidis*	HCSC-Se12	256	8	64	0.25	2	4	16	4
*S. epidermidis*	HCSC-Se31	16	128	128	0.25	1	4	16	4
*S. epidermidis*	HCSC-Se39	16	128	128	0.25	2	2	8	2
*S. epidermidis*	HCSC-Se45	64	64	128	0.125	2	8	16	8
*S. epidermidis*	HCSC-Se47	16	128	128	0.25	2	8	16	8

LNZ: Linezolid; AMK: amikacin; GM: gentamicin; PLZ: plazomicin; FOSF: fosfomycin; IMP: imipenem; MER: meropenem; DOR: doripenem. MIC values determined by broth microdilution assays. Breakpoints according to EUCAST or FDA (plazomicin). * No breakpoints specified by EUCAST for staphylococci.

**Table 2:** Checkerboard synergy testing results for the combinations of linezolid with several antibiotics against staphylococcal isolates: 5 *S. aureus* (Sa) and 5 *S. epidermidis* (Se).

Isolate Number	FICI_min_ (Interpretation) Minimum MICs (mg/L) at Synergistic Point						
	LNZ-AMK	LNZ-GM	LNZ-PLZ	LNZ-FOSF	LNZ-IMP	LNZ-MER	LNZ-DOR
HCSC-Sa3	1 (I)	0.53 (PS)	1 (I)	**0.257 (S)** L(0.25)/F(64) *	0.625 (PS)	0.56 (PS)	0.625 (PS)
HCSC-Sa8	1 (I)	0.75 (PS)	0.75 (PS)	0.75 (PS)	**0.375 (S)** **L(4)/I(32)**	0.75 (PS)	1 (I)
HCSC-Sa11	1 (I)	0.625 (PS)	**0.25 (S)** L(4)/P(0.25) *	0.75 (PS)	**0.5 (S)** **L(8)/I(32)**	0.75 (PS)	0.625 (PS)
HCSC-Sa13	1 (I)	0.75 (PS)	**0.14 (S)** L(0.5)/P(0.25) *	1 (I)	**0.375 (S)** **L(8)/I(32)**	0.75 (PS)	0.75 (PS)
HCSC-Sa14	1 (I)	0.625 (PS)	**0.26 (S)** L(0.5)/P(0.125) *	**0.28 (S)** **L(1)/F(128)**	**0.5 (S)** **L(8)/I(32)**	0.75 (PS)	**0.375 (S)** **L(4)/D(32)**
HCSC-Se12	0.5 (S)	0.625 (PS)	0.75 (PS)	0.625 (PS)	**0.5 (S)** L(0.25)/I(2) *	0.75 (PS)	**0.5 (S)** **L(128)/D(1)**
HCSC-Se31	0.75 (PS)	0.625 (PS)	0.53 (PS)	1 (I)	**0.375 (S)** L(4)/I(2) *	**0.375 (S)** L(8)/M(4) *	**0.25 (S)** L(4)/D(2)*
HCSC-Se39	**0.5 (S)** **L(0.125)/A(128)**	1 (I)	0.625 (PS)	**0.5 (S)** L(0.125)/F(2) *	**0.5 (S)** L(2)/I(0.25) *	0.625(PS)	**0.375 (S)** L(8)/D(1) *
HCSC-Se45	**0.09 (S)** **L(32)/A(8)**	**0.09 (S)** **L(32)/G(16)**	**0.078 (S)** **L(32)/P(0.0156)**	**0.187 (S)** **L(32)/F(0.5)**	**0,187 (S)** L(4)/I(2) *	**0.078 (S)** **L(32)/M(0.25)**	**0.0625 (S)** **L(16)/D(0.25)**
HCSC-Se47	0.75 (PS)	**0.375 (S)** **L(8)/G(64)**	0.75 (PS)	**0.5 (S)** L(8)/F(2) *	**0.315 (S)** L(4)/I(2) *	**0.375 (S)** L(8)/M(4) *	**0.3125 (S)** L(8)/D(0.5) *

LNZ, L: linezolid; AMK, A: amikacin; GM, G: gentamicin; PLZ, P: plazomicin; FOSF, F: fosfomycin; IMP, I: imipenem; MER, M: meropenem; DOR, D: doripenem. FIC_min_: minimum fractional inhibitory concentration index; S: synergy; PS: partial synergy; I: indifferent. Value interpretation: ≤0.5, synergy; >0.5–1.0, partial synergy; >1–<4, indifferent; >4.0, antagonism. Synergistics results are marked with bold characters. * Clinical usefulness, as the concentrations are <SSC values in plasma (SSC, steady state soncentration).

**Table 3:** Log CFU/mL at 24 h for different antibiotics and combinations of 4 *S. aureus* and 4 *S. epidermidis* isolates.

Antibiotic	*S. aureus*				*S. epidermidis*			
	HCSC-Sa3	HCSC-Sa8	HCSC-Sa13	HCSC-Sa14	HCSC-Se31	HCSC-Se39	HCSC-Se45	HCSC-Se47
Control	8.848	8.938	9.362	9.728	9.168	10.476	9.028	9.070
LNZ 1/2MIC	6.255	6.350	7.301	8.653	5.903	8.585	7.977	7.790
AMK SSC	0.845	1.778	5.204	1.000	8.398	4.000	8.176	9.301
GM SSC	0.845	1.778	3.568	9.356	2.477	9.019	5.316	0.845
PLZ SSC	0.845	1.000	0.845	0.845	0.845	0.845	0.845	1.477
FOSF SSC	5.829	6.973	7.970	7.954	8.664	9.015	8.889	9.591
IMP SSC	3.000	8.985	8.602	ND	7.966	ND	6.217	9.423
MER SSC	8.817	9.000	9.267	8.778	8.905	9.025	9.313	9.146
LNZ+AMK	4.921	2.332	**0.845**	2.455	7.628	5.000	3.279 *	**4.916**
LNZ+GM	2.146	0.845	3.125	**5.190**	2.362	**6.699**	0.845 *	2.875
LNZ+PLZ	1.000	0.845	3.622	0.845	0.845	0.845	0.845	4.695
LNZ+FOSF	2.727 *	**3.845**	7.423	8.000	5.450	0.845 *	3.028 *	7.618
LNZ+IMP	3.204	5.243	**5.04**	ND	**3.527**	ND	2.000 *	3.994 *
LNZ+MER	**5.139**	5.394	**7.22**	**4.903**	4.773	**5.041**	2.903 *	2.934 *

LNZ: linezolid; AMK: amikacin; GM: gentamicin; PLZ: plazomicin; FOSF: fosfomycin; IMP: imipenem; MER: meropenem. Combinations were generated using 1/2 of the MIC of linezolid and SSC (steady state soncentration) of the antibiotic in combination. Control: growth in Mueller–Hinton broth without antibiotic. Synergistic results are marked in bold. * Synergistic results coincident with clinically useful ones marked in Table 2.

**Table 4:** Staphylococcal strains included in the study, isolated in an intensive care unit, Madrid, Spain.

Isolate	Clinical Sample	Linezolid Resistance Mechanism
***Staphylococcus aureus*** [17]		
HCSC-Sa3	Bronchial aspirate	*cfr +* ∆Ser 145/His146Tyr (L3)
HCSC-Sa8	Blood	*cfr +* ∆Ser 145/His146Tyr (L3)
HCSC-Sa11	Bronchial aspirate	*cfr +* ∆Ser 145/His146Tyr (L3)
HCSC-Sa13	Bronchial aspirate	*cfr* + ∆Ser 145/His146Tyr (L3)
HCSC-Sa14	Catheter tip	*cfr +* ∆Ser 145/His146Tyr (L3)
***Staphylococcus epidermidis*** [21]		
HCSC-Se12	Catheter tip	*cfr* + Gly2576Thr (rRNA—5 copies)
HCSC-Se31	Blood	Gly152Ser (L3)
HCSC-Se39	Catheter tip	*cfr*
HCSC-Se45	Catheter tip	Gly152Ser (L3) + Asn158Ser (L4)
HCSC-Se47	Catheter tip	Gly2576Thr (rRNA—1 copy)
